# Medical record information disclosure laws and policies among selected countries; a comparative study

**Published:** 2010

**Authors:** Mohammad Hossein Yarmohammadian, Ahmad Reza Raeisi, Nahid Tavakoli, Leila Ghaderi Nansa

**Affiliations:** a Health Management and Economic Research Center, Isfahan University of Medical Sciences, Isfahan, Iran; b Department of Health Services Administration, Health Management and Economic Research Center, Isfahan University of Medical Sciences, Isfahan, Iran; c Medical Records Department, Isfahan University of Medical Sciences, Isfahan, Iran; d Medical Records Department, Tabriz University of Medical Sciences, Tabriz, Iran

**Keywords:** Disclosure of Information, Medical Information, Health Laws and Policies

## Abstract

**BACKGROUND:**

Hospitals have responsibility for responding to legitimate demands for release of health information while protecting the confidentiality of the patient health records. There have always been challenges concerning medical records confidentiality and their disclosure and release type in medical record departments. This study investigated and compared laws and policies of disclosure of health information in Iran and selected countries and tried to identify the differences and the similarities between them.

**METHODS:**

This is a descriptive and comparative study. The scope of study included related laws and policies of disclosure of health information in selected countries such as United States, Australia, England, Malaysia and Iran. Data were gathered from systematic internet search, library resources and communication with health information professionals. Data analysis was done using comparative tables and qualitative method.

**RESULTS:**

Study results showed that legislative institutions of each country have ordained laws and policies concerning disclosure and release of health information and in turn hospitals developed policies and procedures based on these laws. In Iran, however, there are few laws and policies concerning disclosure of health information in the form of formal letters and bylaws. There are no specific written policies and procedures for disclosure of health information in the hospitals.

**CONCLUSIONS:**

It is necessary to develop legitimate and appropriate laws and policies in different levels for information utilization by hospitals, medical universities and others. Meanwhile in all of the selected countries there are ordained limitations for release of health information for protecting health information in regard to patient rights.

Medical records provide evidence to support all aspects of the patient care and it is used by various groups to evaluate and enhance the quality of the care rendered. It also communicates information for facilitating delivery of services, care and treatment to patients. Medical record is also used for research to support decision making, guide performance improvement, and as a legal record when necessary.^1^–^3^ In other words hospital administrators and clinicians would not be able to assure appropriateness and accuracy of health services without accurate, comprehensive and up-to-date medical records.^3^

Medical record departments provide major part of needed information for healthcare systems, and are responsible for protecting privacy and confidentially of patient information. Increased tendency of hospitals for taking advantage of automated systems for medical information, while having no specific and clear rules and regulations, can cause the transfer of information to go out of control and increases the probability of information leak and accessibility of unauthorized people. This is a new challenge for health information managers as well as hospital administrators concerning their new roles and responsibilities.^4^

In Iran, there are no clear and comprehensive rules and regulations in hospitals on how to disclose patient medical records for various applications. Therefore medical record department staff is facing difficult situations for disclosure and transfer of medical records. Some hospitals have established and developed internal policies and procedures which could not adequately protect the confidentiality of patient information and/or satisfy needs of applicants in required circumstances.^5^–^10^ As the first step in striking a proper balance between the personal privacy rights of patients and the informational needs of hospitals and society in general, hospitals have to have a well defined policy and procedures on the use and disclosure of medical information. This policy should limit disclosure to essential purposes.^5^,^6^

In a research done by Health Insurance Portability and Accountability Act (HIPAA) titled the Privacy Rule and Public Health, guided by CDC and the US Department of Health and Human Services, the results expressed that the new national health information privacy standards issued by the US Department of Health and Human Services (DHHS), following the Health Insurance Portability and Accountability Act of 1996 (HIPAA), provide protection for the privacy of certain individually identifiable health data, referred to as Protected Health Information (PHI). Balancing the protection of individual health information with the need to protect public health, the Privacy Rule permits disclosures without individual authorization to public health agencies.^11^

In another research titled Complying with the Health Insurance Portability and Accountability Act privacy standards, results expressed that for purposes of treatment, payment, or routine health care operations the privacy rules limit the use and disclosure of protected health information. It requires the covered organizations to provide advance notice to the public of its policy governing disclosure of protected health information. The covered organizations or entities are required by the standard to secure general client consent to use and to disclose protected health information for treatment, payment, or routine health care operations. They must also obtain specific client authorization to use or to disclose protected health information for all other purposes unless the disclosure is specifically permitted without consent or authorization. In certain situations, considering the circumstances surrounding the disclosure, an organization only needs to obtain client’s agreement to disclose protected health information which may be oral or inferred from the circumstances.^12^

In another research conducted in 2007, assessing the effects of the privacy rule on release of patient information by healthcare organizations, the results showed that the HIPAA privacy rule has had both positive and negative effects on the release of patient information. Although the intention of HIPAA was to protect and promote privacy, security and confidentiality of patient information, it has also had unintended consequences for healthcare facilities. The unintended consequences increased the public misunderstanding about release of patient information, lack of a comprehensive covering policy or regulation defining variations and also enforcement that allows individual institutions to make their own interpretations. Also challenges to health information management professionals in controlling safeguards related to release of information given the transition to electronic health records and the increased involvement of information technology.^13^

A study done by Farzandipoor about disclosure of medical record information for quality monitoring showed that all of the studied hospitals did not follow similar policy for using hospital information. While majority of hospitals utilized and accessed medical records without any limitation and specific regulation, just in a few hospitals disclosure of information required patient’s agreement or hospital managers’ order.^5^

In contrast in another study in the United States of America in 2006 about patient’s privacy and trust, patients’ agreement for allowing researcher to access their medical records illustrated that patients had similar perceptions and tendencies for sharing their medical records and similar recommendations for administering control actions on released information items and procedure of disclosure.^14^

Results of another research in England showed that conditions placed on access to medical records for research purposes raises concerns around negative impacts on research quality and on human subject protection, including privacy due to variation across Research Ethics Boards (REBs). The study suggested that REBs need training in best practices for protecting privacy and confidentiality in health research. A forum for REB chairs concerning confidentially, share concerns and decisions about specific studies that reduce these variation across REBs.^15^

In UK laws, law enforcement personnel and agencies are allowed disclosure to courts and police. Laws emphasize only disclosing the health information required to fulfill and conform with the purpose of the law. If staff had reasons to believe that conforming to a statutory obligation or law to disclose health information would cause serious harm to their client, they should seek legal advice.^16^

The patient consent for disclosure of health information is not necessary for law enforcement officers or agencies, but the disclosing unit must inform the patient. In cases where staffs are concerned that a court order requires disclosure of sensitive information that is not critical to the case in question, they may raise ethical concerns with the judge or presiding officer. In cases where disclosure to police is obligatory it does not require the consent of the patient. In the absence of a requirement to disclose, there must be either explicit patient consent or robust interest justification.^16^

This research was done to review policies and laws related to disclosure of medical record information among selected countries and tried to identify the similarities and the differences between them in order to develop and adopt related policies and laws for Iranian hospitals.

## Methods

This was a descriptive-comparative study done from February 2008 through October 2009. The research resources included written and electronic documents and records. Data were gathered regarding health information disclosure laws and policies related to disclosure of health information in five countries namely United States, Australia, England, Malaysia and Iran. The reason for selection of United States, Australia and England is their advancement and long history and experience of these countries in the field and establishment of medical record programs and departments. Malaysia is an Islamic country that in recent years has had rapid growth and development in the field of health information and communication technology. The research information were gathered from the Department of Health and Human Services (DHHS), Health Care Financing Administration (HCFA) and American Health Information Management Association (AHIMA) in USA, British Medical Association (BMA), Department of Health in England (DH), National Health Services (NHS), Department of Health and Ageing, Human Research Ethical Committee (HREC), Health Information Management Association of Australia (HIMAA), Malaysia Medical Council (MMC), Department of Health of Malaysia and the Iran Ministry of Health and Medical Education academic medical centers.^6^,^16^–^24^

The study data were gathered from April until September 2008 in six months period with using key words and terms like disclosure of Information, medical information, health laws and policies taking advantage of systematic electronic search via internet in databases specializing in the field of confidentiality and disclosure of health and medical information such as AHIMA, HIPAA, AMA, CDC, AHA, HIMAA,^11^,^16^,^17^,^21^,^25^–^29^ and official websites of medical records associations of selective countries and library resources, review of hard and electronic written documents and records in Iran Ministry of Health and Medical Education, HBI^6^ and related departments of Health. Also communicated with professionals and surveyed organizations via e-mail namely Victoria Monahan, Privacy Contact Officer of Legal and Legislative Services Branch Department of Health UK; Lyn Williams, Team Leader, Education Services, Health Information Management Association of Australia Ltd; Jill Petrie, Office Manager of UK; Dr Norakma; Dr Zuhaida; Cik Daisy; and Dr S Selvaraju of Malaysia. Data analysis was performed with comparative tables and qualitative analysis method.

## Results

The study results showed that in the selected countries, patients have the right to access their own medical records unless the information would cause serious harm to them or another person or may have an adverse impact on their physical and/or mental health. This right in UK laws is also reserved for former residents of the country who live outside the UK so that they have the right to apply for access to their former UK health records.

United States, UK and Australia have adopted special security measures for patient access to medical records. When a patient requests for access to his/her health information, medical records managers must first ensure the applicant identification and next the release of health information must be in consultation with a related physician; so after the physician’s confirmation, health information would be released to the patient. When a patient requests direct inspection of the health records, this access should be supervised by the attending staff of health information management professionals and/or the department manager. When patient access to their medical record takes place, the attendance of the medical record or manager must not consulate the patient in regard to their medical record contents and query on behalf of patient and the patient must be referred to his or her physician. In Iran, in cases of incurable diseases it is recommended that the patients not to be allowed to access their medical records without the permission of his or her physician.

Disclosing confidential patient health information for care continuance to clinical specialist has been clarified for researcher in selected countries except in Malaysia (Table 1).

**Table 1:** Components of information disclosure to patient or family members

Components of disclosure to medicai record users	USA	UK	Australia	Malaysia	Iran
Components of disclosure to patient:					
1-Security safeguard and health information confidentiality	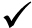	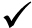	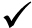	-	-
2-Credible tool for identify applicant person before access to medical record	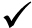	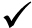	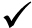	-	-
3-Survey of original record supervised by patient in attendance of staff for guidance of record and answer to questions of patient and safeguard security of information	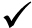	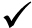	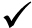	-	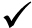
4-Confirmation of the access to information with discretion of a physician	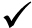	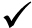	-	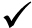	-
5-Definition of non-access cases	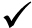	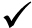	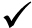	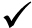	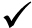
6-Inform to patient in field of refuse of access request to record	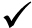	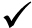	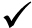	-	-
7-Identify accountability to requests	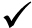	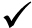	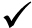	-	-
8-Receive charges for release of information Disclosure to the patient family members:	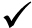	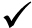	-	-	-
Disclosure to the patient family members:					
1-Disclosure to the patient family members for payment expenditures of patient	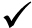	-	-	-	-
2-Disclosure to the patient family members when the patient is minor	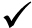	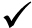	-	-	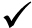
3-Disclosure to the patient family members in cases of incompetence or incapacity	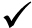	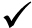	-	-	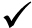
4-Disclosure to the patient family members in compassionate grounds	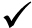	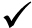	-	-	-
5-Disclosure to the patient family members for interest of patient	-	-	-	-	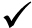

As given in table 1 in Australia disclosure of health information to the patient family members on a compassionate ground was done in the emergency situations or in cases where a patient has passed away. In the United States, disclosure of health information to a family member was done in order to notify or assist in notifying a family member of the location, general condition or death of the patient. In UK and Iran there are defined policies for disclosure of health information to a patient family member when the patient is minor, emancipated minor or in cases of incompetence or incapacitation whether temporary incapacitation or permanent incapacitation. In the United States in the case of under aged minor, his or her parents makes authorization decisions on behalf of the child and in cases of lengthy or permanent incapacity a legal guardian for the patient may be appointed by the court or a person may grant power of attorney to another person, which authorize a designee to act on behalf of the person who is incapacitated. In cases of temporary incapacity, health care provider should discuss the basic facts of patient condition and the emergency plan to family members. (Table 2)

**Table 2:** Components of information disclosure to social users of medical records

Components of disclosure to medicai record users	USA	UK	Australia	Malaysia	Iran
Disclosure of information for quality monitoring purposes:					
1-Disclosure of information for monitoring quality purposes without patient authorization	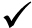	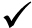	-	-	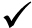
2-Adoption of security measures when audit do by persons outside of organization	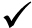	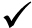	-	-	-
Disclosure of information for educational purposes:					
1-Use of anonymous data for teaching without patient consent	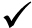	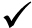	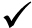	-	-
2-Adoption patient authorization when they use of identification data of patient	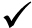	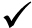	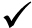	-	-
3-Adoption of security measures for safeguard of data when answer to requests of students	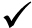		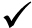	-	-
Disclosure of information for research purposes:					
1-Disclosure of information for research purposes without patient authorization	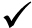	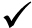	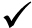	-	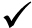
2-Approve proposal in Human Research Ethics Committee when they use identification data	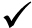	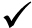	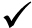	-	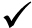
3-Existence of mechanisms for de-identification data for research purposes	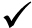	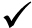	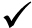	-	-
Disclosure of information for administrative purposes:					
1-Distention of access to medical record on basis need to know for administrative purposes without patient authorization	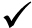	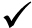	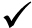	-	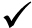
Disclosure of information for payment purposes:					
1-Disclosure of information to insurance company with written authorization of patient	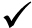	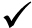	-	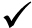	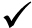
Disclosure of information to attorneys:					
1-Release of information to attorneys with authorization of patient or her/his representative, valid subpoena or court order	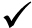	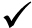	-	-	-
2-Access of legal counsel of hospital to information without patient authorization	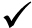	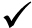	-	-	-
3-Reviewing authorizations submitted by attorneys per health information professional	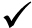	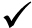	-	-	-
4-Disclosure of health information for workers compensation	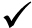	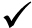	-	-	-

Findings in table 2 indicated that disclosure of health information for quality monitoring has been clarified in selected countries except Malaysia and disclosing health information for internal auditors don’t need patient’s consent. In Malaysia, there isn’t any related clear policy.

In United States, UK and Australia, written policies exist for disclosing health information for educational purposes. However these policies are not clearly defined in Iran and Malaysia. In the United States, United Kingdom and Australia disclosure and use of confidential health information for educational needs does not require patient’s authorization except in cases where identification of patient would defeat the purpose of the training or the material has critical importance to health system the consent of the patient has been obtained.

United States, United Kingdom and Australia have adopted special security measures for accountability purposes to the student requests to protect patient data and no laws in this regard were found in Iran and Malaysia.

Written policies existed for disclosure of health information for research objectives but it must be approved by the Human Research Ethics Committees (HREC) in USA and Australia which has responsibility for surveying confidential matter.

United States, UK and Australia have adopted mechanisms for data de-identification for research purposes and had defined situations where disclosing health information required patient authorization.

United States and Australian policies for de-identification situations had provided for proposal to submit to HREC expressed with details.

Disclosure of health information is clarified for administrative purposes in the selected countries, where disclosing health information did not require patient authorization for this purpose. Australia has precise laws for disclosing health information used for management of health services activities. Health care organizations consider the questions that survey likelihood of the risk or burden to patient or risk of breaching the confidentiality and necessity of access to patient information. If answers to the questions were contrary with patient interests, the request must be approved by the Human Research Ethics Committee. In Iran policies exist only for administrative purposes that emphasis use of anonymous data that does not require patient consent.

Disclosing health information for payments clarified for researchers in selected countries except in Australia. Use of such information for payment purposes is considered impersonal, in other words it requires authorization of the patient or the patient legal representative. Disclosing health information to attorneys for research is clarified in the United States and UK but this study did not find information in Australia, Iran and Malaysia in this regard. In the United States and UK for release of health information to attorneys required written authorization of the patient or the patient legal representative or a valid subpoena.

In United States the hospital legal counsel does not require patient authorization to obtain access to specific record. In UK if disclosure of health information is to be in the public interest even without patient consent legal counsel can access the medical record; of course this public interest should be expressed by court. In United States and UK when confronted with challenges for release of health information to attorneys, they must get consult from hospital legal counsel and facility risk manager. British Medical Association grant this right where staff believe that medical records contains sensitive information, hospitals must not disclose and can object to the judge or presiding officer. (Table 3)

**Table 3:** Components of disclosure of health information to law enforcement personnel or agency

Components of disclosure to law enforcement personnel or agency	USA	UK	Australia	Malaysia	Iran
1 -Disclosure is required by law or court order or subpoena	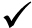	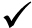	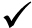	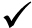	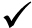
2-Disclosing confidential information of patient to law enforcement personnel or agency without patient authorization	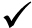	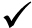	-	-	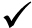
3-Disclosure to identify or locate a suspect, fugitive, material witness or missing person	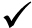	-	-	-	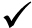
4-Disclosure to alert law enforcement personnel or agency from nature or location or identity of the perpetrator of the crime	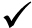	-	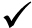	-	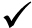
5-Disclosure of mandatory reporting requirement	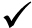	-	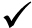	-	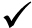

As given in table 3 disclosure of health information must be strictly in accordance with the terms of a court order or subpoena to law enforcement personnel or agencies in selected countries. This type of disclosure does not take place without the consent of the patient.

In United States, UK and Iran, the hospital may disclose health information to law enforcement officials without authorization of the patient when one of the following conditions is met:

- Disclosure is required by law or is made in compliance with a court order.
- Disclosure is made in response to a legal activity to identify or locate a suspect, fugitive, material witness or missing person, alert law enforcement of the individual who is or is suspected to be a victim of a crime and the suspicion that a patient death may have resulted from criminal conduct.
- Disclosure of mandatory reporting requirement e.g. reporting of births and deaths, reporting of communicable disease and cancer.

Some of the US states must report abuse of childs/adults, domestic violence and special wounds.

## Discussion

Results showed that legislative institutions of each country have ordained laws and policies concerning qualification and people interests and in turn hospitals developed policies and procedures based on these laws. Meanwhile in all of the selected countries there are ordained limitations for release of health information for protecting health information in regard to patient rights. In Iran, however, there are sporadic laws and policies concerning disclosure of health information in the form of formal letters and bylaws. However there are no specific policies and procedures for disclosure of health information.

Regarding health information disclosure to patient or family members, there is similarity in policy and procedure between United States and United Kingdom. They have complete policies about disclosure of information to patient, but other three countries (Australia, Malaysia and Iran) policies related to disclosure of health information to patient or family members are inadequate. There are also same results about disclosure to other users of medical records among the countries.

Findings generally showed that there are similar policies between USA and Iran about disclosure of health information to law enforcement personnel or agencies. Other countries like United Kingdom, Australia and Malaysia have limited and partial policies and procedures.

In Iran, legal authorities should approve specific policies and laws in regard to using medical records information for patients or family members and other users of medical records, because there is major shortcoming in these fields, but findings showed that there are rigid and strong policies for legal users and law enforcement agencies.

Despite the fact that patient or his/her legal representative agreement is common requirement for disclosure of information for repayment purposes in all the selected countries, there isn’t any clear and specific policy and procedure in Iran in this regard. There is just a general and brief recommendation for disclosure of medical records information to the patient or his or her legal representative. There is correlation between findings of current research and some other researches which showed an ambiguity about disclosure of information for insurance companies in Iranian laws.^4^,^5^,^7^–^9^,^30^,^31^ In Iran disclosing information to insurance companies is subject to approved contracts. Insurance officers are required to investigate and prepare compatible medical records to support patients insurance. They should also compare records and health invoices to their own insurance terms and conditions. Lack of cooperation between hospitals especially the medical records departments and insurance companies has actually resulted in difficulty for refunding treatment expenses. Also lack of specific and clear policies despite responding to applicant requests causes violation of patient rights and neglecting the confidentiality of medical records.

## Conclusions

In conclusion, legal authorities should approve specific policies and laws in regard to using medical records information for patients or family members and other users of medical records according to national and social circumstances of Iran. It is also recommended that further research should be conducted to do comparative studies about disclosure of health information in teaching hospitals and challenges concerning security and confidentiality of medical records.
